# Histone methyltransferase KMT2A promotes pulmonary fibrogenesis via targeting pro‐fibrotic factor PU.1 in fibroblasts

**DOI:** 10.1002/ctm2.70217

**Published:** 2025-01-30

**Authors:** Wenting Lyu, Hui Wang, Tong Ji, Ling Liu, Haoran Chen, Li Fan, Guanning Zhong, Naihui Wan, Suwan Chen, Jingyu Chen, Hourong Cai, Hongyang Xu, Dongjin Wang, Jinghong Dai

**Affiliations:** ^1^ Department of Pulmonary and Critical Care Medicine The Affiliated Drum Tower Hospital of Nanjing University Medical School Nanjing Jiangsu China; ^2^ Development and Related Disease of Women and Children Key Lab of Sichuan West China Second University Hospital Sichuan University Chengdu China; ^3^ Department of Respiratory and Critical Care Medicine Shanghai Pulmonary Hospital School of Medicine Tongji University Shanghai China; ^4^ Department of Pulmonary and Critical Care Medicine The Second Affiliated Hospital of Soochow University Suzhou China; ^5^ Department of Respiratory and Critical Care Medicine The Second People's Hospital of Yibin Yibin Sichuan China; ^6^ Department of Critical Care Medicine The Affiliated Wuxi People's Hospital of Nanjing Medical University Wuxi People's Hospital Wuxi Medical Center Nanjing Medical University Nanjing China; ^7^ Department of Cardiothoracic Surgery The Affiliated Drum Tower Hospital of Nanjing University Medical School Nanjing Jiangsu China

**Keywords:** epigenetics, fibroblast, histone methylation, idiopathic pulmonary fibrosis

## Abstract

**Background:**

Idiopathic pulmonary fibrosis (IPF) is a fibrotic disease driven by both environmental and genetic factors. Epigenetics refers to changes in gene expression or cellular phenotype that do not involve alterations to DNA sequence. KMT2A is a member of the SET family which catalyses H3K4 methylation.

**Results:**

Through microarray and single‐cell sequencing data, we discovered KMT2A‐positive fibroblasts were increased in IPF lung tissues. KMT2A level was increased in IPF and bleomycin‐induced pulmonary fibrosis mice lung tissues collected in our centre. Mice with AAV6‐induced KMT2A knockdown in fibroblast showed attenuated pulmonary fibrosis after bleomycin treatment. Bioinformation also revealed that transcription factor PU.1 was a target of KMT2A. We demonstrated that PU.1 levels were increased in IPF tissues, bleomycin‐induced mice lung tissues and primary fibrotic fibroblasts. KMT2A knockdown decreases PU.1 expression in vitro while KMT2A overexpression induces PU.1 activation. PU.1 fibroblast‐specific knockout mice showed attenuated lung fibrosis induced by bleomycin. Furthermore, we demonstrated KMT2A up‐regulated PU.1 in fibroblasts by catalysing H3K4me3 at the promoter of the PU.1 gene. The KMT2A transcription complex inhibitor mm102 treatment attenuated bleomycin‐induced pulmonary fibrosis.

**Conclusion:**

The current study indicated that histone modification participates in the pathogenesis of IPF and KMT2A may have the potential to be a therapeutic target of IPF treatment.

**Key points:**

- KMT2A plays a role in pulmonary fibrogenesis.
- KMT2A regulates PU.1 transcription in fibroblasts through H3K4me3 at promoter.
- KMT2A inhibitor attenuates pulmonary fibrosis in mice.

## Graphical abstract

This study demonstrated how KMT2A–WDR5 regulate pro‐fibrotic factor PU.1 through catalysing H3K4me3 at the promoter. We suggested both knocking down KMT2A and inhibiting KMT2A transcription complex could attenuate lung fibrosis, which indicating new therapeutic strategy for clinical practice.

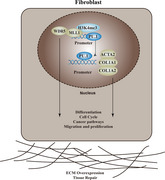

## 1 INTRODUCTION

Idiopathic pulmonary fibrosis (IPF) is a chronic, progressive fibrotic lung disease characterised by worsening shortness of breath and respiratory failure.^1^ In recent years, the incidence of IPF has been steadily increasing due to factors including aging population and environmental influences like air pollution, with a high mortality rate. The median survival time for diagnosed patients is only 3–4 years.^2^ Currently, anti‐fibrotic drugs such as pirfenidone and nintedanib are recommended as the best treatment options by international guidelines. Pirfenidone is a pyridine compound which can inhibit the differentiation of fibroblasts mediated by transforming growth factor beta (TGFβ) and reduce the synthesis and deposition of extracellular matrix in vitro.^3^ Nintedanib is a tyrosine kinase receptor inhibitor that blocks the pro‐fibrotic effects of multiple growth factors mediated by tyrosine kinases, thereby slowing the progression of IPF.^4^ However, these drugs can only slow down the deterioration of lung function and cannot reverse the ongoing pathological process of lung fibrosis.^5^, ^6^ Identifying the key mechanisms underlying the continuous progression of IPF and exploring targeted treatment strategies based on these mechanisms is a crucial direction for addressing the challenges in IPF therapy.

IPF is a disease caused by both environmental and genetic factors, with epigenetic modification mechanisms playing a key role in regulating pro‐fibrotic genes. Research has identified changes in microRNA expression,^7^, ^8^ alterations in the methylation status of promoters of specific disease‐related genes^9^, ^10^, ^11^ and significant methylation changes in many CpG islands within IPF lung tissue, all of which are associated with the onset and progression of IPF, providing evidence for the role of epigenetic regulation.^12^ As previously mentioned, IPF presents significant therapeutic challenges, making the exploration of new therapeutic pathways and targets for its development and progression of great value. In recent years, clinical trials targeting epigenetic factors have been conducted for certain diseases, yielding promising results. Exploring and attempting therapeutic strategies targeting epigenetic factors may offer new choice for the treatment of IPF.

Histone modification is an important epigenetic mechanism in the pathogenesis of pulmonary fibrosis, with some studies already reported. Histone lysine methyltransferase 2A (KMT2A) is a member of the SET family responsible for catalysing H3K4 methylation, exerting its histone modification catalytic function through the SET domain.^13^ KMT2A is involved in the differentiation, cell cycle regulation, proliferation and self‐renewal of various mesenchymal‐derived cells, playing a crucial role in organ repair and regeneration.^14^ In recent years, the function and target genes of KMT2A in fibrotic diseases have gained attention, with different downstream targets performing various functions, making it a valuable research subject. However, its role in pulmonary fibrosis is less studied. One potential downstream target of KMT2A, PU.1, is a key factor in the gene expression regulatory network associated with cellular differentiation, with its expression level forming a positive feedback loop that promotes differentiation, and it also regulates processes such as cell senescence, cell cycle and autophagy. Recent studies have found that abnormal activation of PU.1 was associated with the development of various fibrotic diseases.^15^, ^16^ In the skin fibroblasts of patients with systemic sclerosis, knocking out the PU.1 gene can down‐regulate α‐SMA expression, and knocking out PU.1 in mouse fibroblasts can alleviate fibrosis in the liver and skin of model animals.^16^ However, the specific function of PU.1 in pulmonary fibrosis fibroblasts remains unclear.

Based on the current research landscape, this study aims to elucidate the molecular mechanisms by which KMT2A histone methylation modification targets the regulation of PU.1 expression, revealing the network of transcription factor PU.1 and downstream pro‐fibrotic gene expression. This research holds the potential to provide new therapeutic targets for the treatment of IPF.

## 2 METHODS

### 2.1 Ethics statement

A total of eight patients with IPF and eight patients with cancer adjacent normal lung tissues were included as control. Left lower lung of IPF patients who underwent lung transplantation were obtained for the research. Informed written consents were signed by all participants involved. IPF diagnoses were made by pulmonologists following the 2022 American Thoracic Society guidelines.^17^ Lung tissues from both the IPF and control groups were obtained from Nanjing Drum Hospital and Wuxi People's Hospital. All animal and human experiments were approved by the Ethics Committee of Nanjing Drum Tower Hospital (2022‐067‐02).

### 2.2 Bioinformatic analysis

Microarray (GSE24206, 17 IPF samples and six healthy donors) and single‐cell (GSE128033, five IPF samples and five healthy donors) were chosen for further analysis. Datasets were downloaded from Gene Expression Omnibus website. All the analysis were conducted using R software 4.3. The codes are all available on the link (https://github.com/lyuwenting/wenting). Cut‐tag analysis was finished by Novogene Cloud Tool.

### 2.3 Mice

All experimental animal procedures were conducted in accordance with humane animal care standards with approval from the Nanjing Drum Tower Hospital Ethics Committee. Male C57/B6 mice (25–30 g, 8 weeks old) were intratracheally administered either saline or 5 mg/kg bleomycin (#S1214; Selleck) dissolved in saline. The control group was treated with 50 µL of sterile saline in the same manner. Mice were sacrificed on day 21, and lung tissues were collected for further analysis. Spi1^fl/−^ mice (B6/JGpt‐Spi1^em1Cflox/^Gpt; GemPharmatech) and Col1a2‐cre/ERT (B6.Cg‐Tg (Col1a2‐cre/ERT,‐ALPP) 7Cpd /2J; Jackson Laboratory) mice were crossed to obtain Spi1^flfl−^ Col1a2‐cre/ERT mice. Tamoxifen (100 mg/kg) was applied intraperitoneally for five times on mice at an age of 4 weeks. The AAV‐6 vectors mediating KMT2A knockdown with the Col1a2 promoter were constructed by Genechem, Shanghai. Mice were randomly assigned to groups, with six mice per group. The vectors were administered intratracheally to 8‐week‐old mice. A bleomycin‐induced model was established after 3 weeks. Lung tissues were collected on the 21st day following bleomycin instillation. For mm102 inhibition in vivo, the mice were randomised into groups and there were six mice in every group. After 14 days, the bleomycin was intratracheally instilled, mm102 (2.5 mg/kg) was injected intraperitoneally seven times daily. Lung tissues were harvested at the 21st day from bleomycin instillation. The measurement of hydroxyproline concentration was finished according to the instructions provided in the manual (#BC0250; Solarbio).

### 2.4 Primary fibroblasts isolation and culture

Primary fibroblasts from mouse and human lungs were isolated and cultured. Lung tissues were cut into 5 mm^3^ (1 mm^3^ for mice) fragments. Tissues were digested with 10 mL of Collagenase I (0.2%; Sigma) at 37°C, shaking for 40 min. Digested products were terminated with 10 mL of complete culture medium and centrifuged at 1500×*g* for 5 min. Tissues were resuspended in PBS containing 15% foetal bovine serum after discarding the supernatant and centrifuged at 1500×*g* for 5 min. After washing for three times, tissues were resuspended in 10 mL of DMEM medium containing 5% foetal bovine serum and transferred to a 10 cm tissue culture dish. Cells were placed in a 37°C, 5% CO_2_ tissue culture incubator. Fibroblasts were observed growing out of the tissue fragments after 6–8 days, developing into a near confluent monolayer of cells after 3–4 weeks. Experiments were conducted on cells between passages 3 and 8.

### 2.5 Small‐interfering RNA‐mediated gene knockdown

MRC‐5 cell line (#GNHu41) were purchased from National Collection of Authenticated Cell Cultures. The small‐interfering RNA (siRNA) targeting human KMT2A mRNA (NM_001197104.2) was synthesised with the sense sequence 5′‐GCACTGTTAAACATTCCACTT‐3′. KMT2A siRNA and scrambled siRNA were purchased from Ruibo (Shanghai, China). Double‐stranded RNAs (50 nM) were transfected into cells using RNAi Max (Invitrogen), with the control siRNA applied at the same concentration.

### 2.6 Plasmid transfection and inhibitor application

Plasmid pMSCV‐FlagMLL‐pl‐ENL was purchased from Addgene (#20873). The vector (3 µg/3 × 10^5^ cells) was transfected into cells by lipofectamine 2000 (Invitrogen). TGFβ (20 ng/mL) was applied 24 h after the transfection. Cells were collected 48 h after TGFβ stimulation. KMT2A inhibitor mm102 (50 µM; Selleck) was applied 12 h before TGFβ.

### 2.7 Histopathological analysis

Histopathological analysis of formalin‐fixed lung biopsy tissues from IPF patients and mice was conducted using haematoxylin and eosin (H&E) and Masson's trichrome staining. The percent of fibrotic area analysis was performed using ImageJ. Primary antibodies of immunohistochemistry were KMT2A (ab92486) and PU.1 (2266; Cell Signaling Technology, USA).

### 2.8 Quantitative real‐time PCR

Total RNA was isolated from frozen cells using TRIZOL Reagent (R401‐01; Vazyme, China). Real‐time PCR was performed as described previously.^18^

### 2.9 Western blot

Western blot analysis was performed as described previously.^18^ Primary antibodies were anti‐KMT2A‐C‐terminal (14197; CST), anti‐KMT2A‐N‐terminal (14689; CST), anti‐αSMA (19245; CST), Collagen I (66761‐1‐Ig; Proteintech), P4HB (ab2792; Abcam), PU.1 (2266; CST), WDR5 (13105; CST), H3K4me3 (9751; CST) and β‐actin (sc‐47778; Santa Cruz). The secondary antibodies used were goat anti‐rabbit (Ab6721) and rabbit anti‐mouse (Ab6728) antibodies (Abcam).

### 2.10 Immunofluorescence

Lung tissues were fixed in 4% paraformaldehyde and embedded in paraffin, while cells were cultured on coverslips for staining. Tissue sections were incubated with primary antibodies at 37°C for 1 h, followed by overnight incubation at 4°C. Secondary antibodies were applied for 1 h at 37°C. After incubation, sections were examined under light microscopy, with negative controls excluding the primary antibody. Nuclei were counterstained with DAPI, and images were captured using a confocal laser scanning microscope. For statistical analysis of double‐staining positive cells, confocal images were randomly captured with an FV3000 confocal microscope (OLYMPUS), with at least three fields of view per section. The primary antibodies used included anti‐KMT2A (sc‐374392; Santa Cruz), anti‐PU.1 (2266; Cell Signaling Technology), anti‐P4HB (ab2791; Abcam) and anti‐αSMA (19245; CST).

### 2.11 CUT&RUN

MRC‐5 cell lines were used in the assay. All experiments were conducted according to the instructions provided in the manual (Hyperactive pG‐MNase CUT&RUN Assay Kit for PCR/qPCR; Vazyme). Cells were collected and mixed with ConA beads. Anti‐H3K4me3 (9751; CST) antibody was added and incubated at 4°C overnight. The mixture was incubated with pG‐MNase Enzyme for fragmentation. FastPure gDNA Mini Columns were used then to collect the fragmented DNA for PCR experiments.

### 2.12 CUT&Tag

MRC‐5 cell lines were used in the assay. All experiments were conducted according to the instructions provided in the manual (Hyperactive Universal CUT&Tag Assay Kit for Illumina; Vazyme). Cells were collected and mixed with ConA beads. Anti‐H3K4me3 (9751; CST) antibody was added and incubated at 4°C overnight. The mixture was incubated with pA/G‐Tnp and TTBL for fragmentation. The fragmentation products were incubatedwith Proteinase K and DNA Extract Beads to collect DNA. After library preparation and purification with the Illumina Kit, sequencing was performed on the Novaseq‐SE50 platform.

### 2.13 Statistical analysis

All assays were conducted in triplicate and repeated at least three times. Results are presented as mean ± standard deviation (SD). Significant differences between means compared with the control were assessed using Student's *t*‐test, with *p* < .05 considered statistically significant.

## 3 RESULTS

### 3.1 KMT2A was highly expressed in fibroblasts within fibrotic lung tissue

**FIGURE 1:**
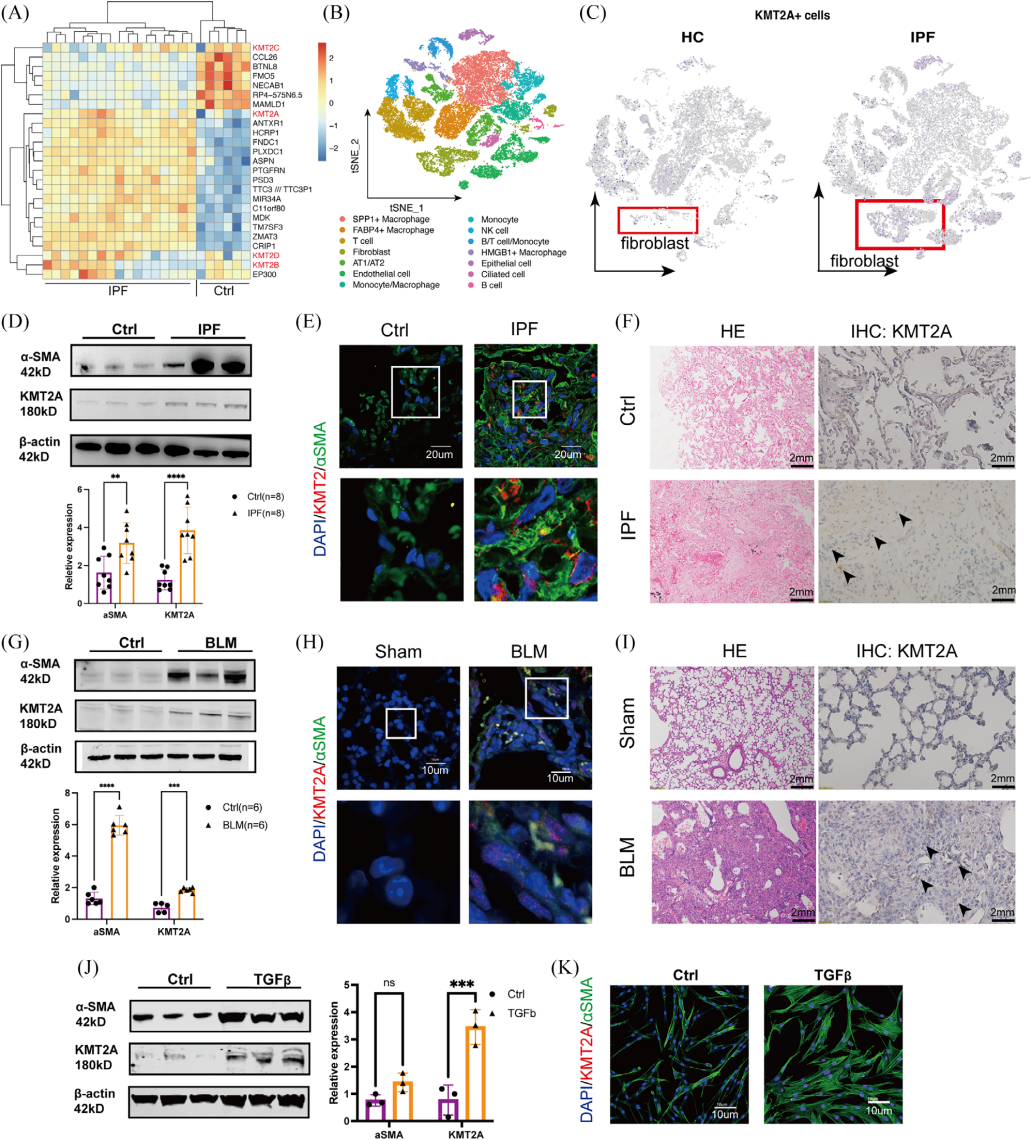
KMT2A was highly expressed in fibroblasts within fibrotic lung tissue. (A) KMT2A was up‐regulated in IPF tissues in DNA array dataset GSE24206. (B) The clustering results of GSE128033 included 37 065 cells in total and the cells were clarified into 14 clusters. (C) Feature plot of KMT2A expression in single‐cell dataset showed increased KMT2A+ fibroblasts in IPF group. (D) Western blotting images and relative expression of α‐SMA and KMT2A in human primary lung fibroblast showed KMT2A was up‐regulated in fibrotic fibroblasts. (E) Immunofluorescent staining of α‐SMA and KMT2A showed more KMT2A‐positive fibroblasts in IPF tissue. (F) Immunohistochemical staining of IPF tissue sections showed an increased number of KMT2A‐positive cells in the IPF tissues. (G) Western blotting images and relative expression of α‐SMA and KMT2A in mice primary lung fibroblast. (H) Immunofluorescent staining of α‐SMA and KMT2A showed more KMT2A‐positive fibroblasts in bleomycin‐induced fibrotic tissue. (I) Immunohistochemical staining showed an increased number of KMT2A‐positive cells in the lung tissues of BLM mice compared with the control group. (J) Western blotting images and relative expression of α‐SMA and KMT2A in TGFβ‐stimulated MRC‐5 cells showed KMT2A was up‐regulated in fibrotic fibroblasts derived from bleomycin‐induced mice model. (K) Immunofluorescent staining of α‐SMA and KMT2A in TGFβ‐stimulated MRC‐5 cells showed up‐regulated KMT2A expression. *p < .05, **p < .01, ***p < .001, ****p < .0001.

To identify potential epigenetic abnormalities in IPF, we selected the GSE24206 microarray dataset from the GEO database for expression profile analysis. Notably, the gene encoding lysine methyltransferase 2A (KMT2A), also known as Mixed Lineage Leukaemia 1 (MLL1), was significantly up‐regulated in the IPF samples. However, other members of the KMT family, such as KMT2B, KMT2C and KMT2D, showed no significant expression differences in this dataset (Figure 1A). Single‐cell RNA sequencing (GSE128033) showed a cluster of KMT2A+ fibroblast in IPF tissues (Figure 1B,C). We validated the expression of KMT2A was elevated in IPF‐derived primary fibroblasts (Figure 1D). Immunohistochemical staining of IPF tissue sections showed an increased number of KMT2A‐positive cells in the IPF tissues. Using an α‐SMA antibody to label activated fibroblasts and co‐staining with KMT2A, we observed that the expression level of KMT2A in the nuclei of fibroblasts was higher compared with the control group (Figure 1E,F). We found that in the bleomycin‐induced lung fibrosis mice model, the levels of α‐SMA and KMT2A were also notably up‐regulated in primary fibroblasts (Figure 1G). Immunohistochemical staining showed an increased number of KMT2A‐positive cells in the lung tissues of BLM mice compared with the control group. Immunofluorescence co‐localisation on paraffin‐embedded lung tissue sections from mice revealed that after using α‐SMA antibody to label fibroblasts and co‐staining with KMT2A, the expression of KMT2A was higher in the nuclei of fibroblasts (Figure 1H,I). Also, KMT2A was up‐regulated in TGFβ‐induced myofibroblasts (Figure 1J,K).

### 3.2 KMT2A knockdown protected mice from bleomycin‐induced lung fibrosis

**FIGURE 2:**
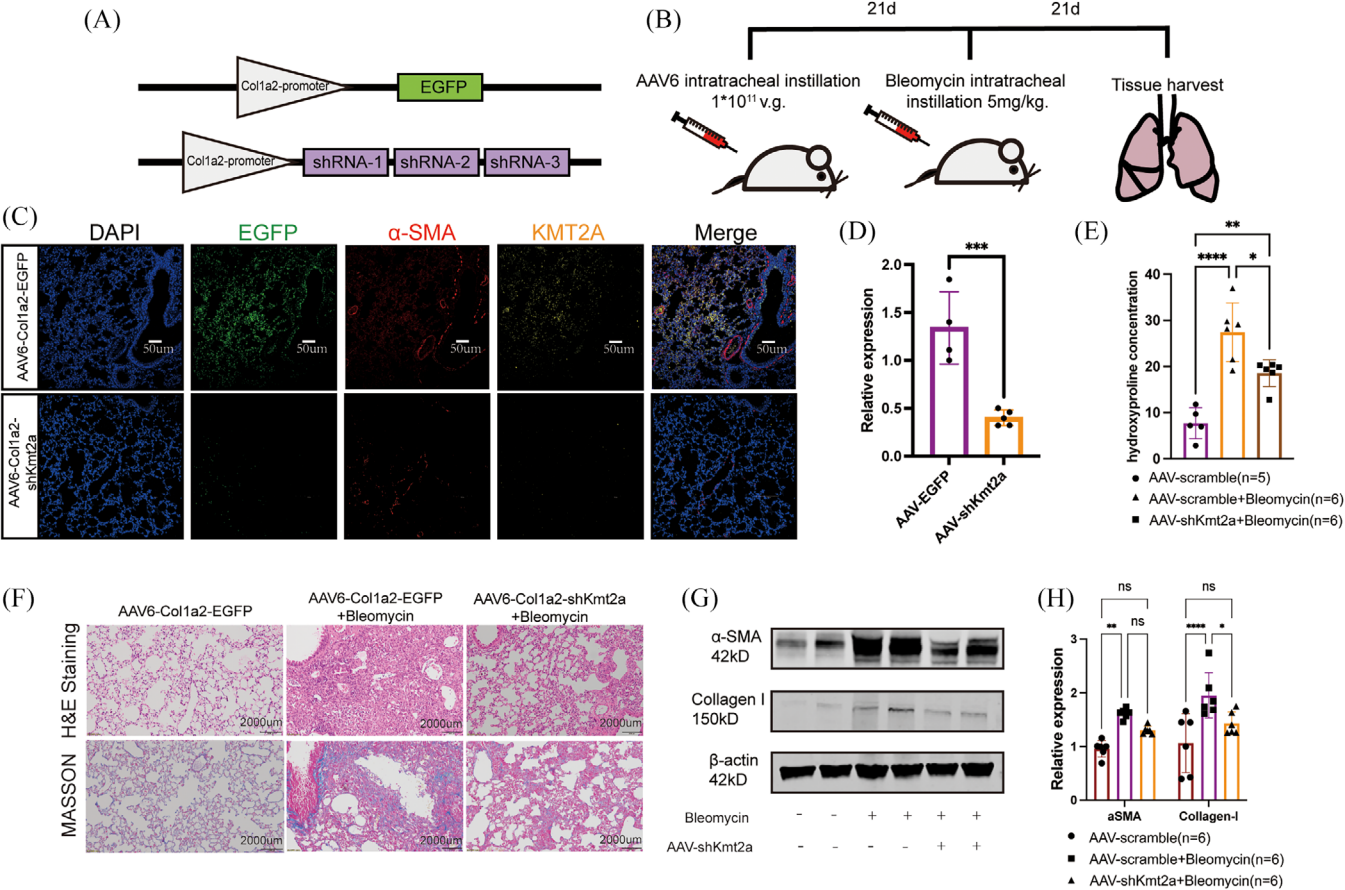
KMT2A knockdown protected mice from bleomycin‐induced lung fibrosis. (A) The construction strategy of AAV6‐Col1a2‐sh‐Kmt2a. (B) Designing of mice experiments for AAV infection. (C) Immunofluorescent staining of EGFP, SMA and KMT2A showed the AAVs were successfully infected and KMT2A was knocked down in fibroblasts. (D) Quantitative realtime PCR showed KMT2A was knocked down at mRNA level. (E) The measurement of hydroxyproline concentration indicates that KMT2A knockdown alleviates pulmonary fibrosis in mice (µg/mL). (F) Haematoxylin and eosin (HE) staining and Masson trichrome staining. (G and H) Western blotting images and relative expression of α‐SMA and Collagen I were reduced in mice lung tissues infected with AAV6‐sh‐Kmt2a. *p < .05, **p < .01, ***p < .001, ****p < .0001.

To validate the potential role of KMT2A in the development of pulmonary fibrosis, we utilised AAV6 (adeno‐associated virus serotype 6) carrying three segments of Kmt2a gene shRNA sequences and designed a Col1a2‐specific promoter to achieve higher efficiency of infection in fibroblasts (Figure 2A,B).Western blotting results indicated that the expression of Collagen I was significantly down‐regulated in the lungs of mice treated with AAV6‐shKmt2a+BLM, while α‐SMA showed a decreasing trend but did not reach statistical significance (Figure 2G,H). The measurement of hydroxyproline concentration indicates that KMT2A knockdown alleviates pulmonary fibrosis in mice compared with the control group (Figure 2E). H&E staining and Masson trichrome staining revealed that mice treated with AAV6‐shKmt2a had significantly lower levels of pulmonary fibrosis compared with those treated with AAV6‐EGFP following bleomycin modelling (Figure 2F). These findings suggest that the expression of KMT2A in lung fibroblasts plays a regulatory role in the development of pulmonary fibrosis, and its deficiency can alleviate fibrosis in the mouse disease model, indicating that KMT2A could be a potential therapeutic target for pulmonary fibrosis.

### 3.3 Transcription factor PU.1 was a target of KMT2A

**FIGURE 3:**
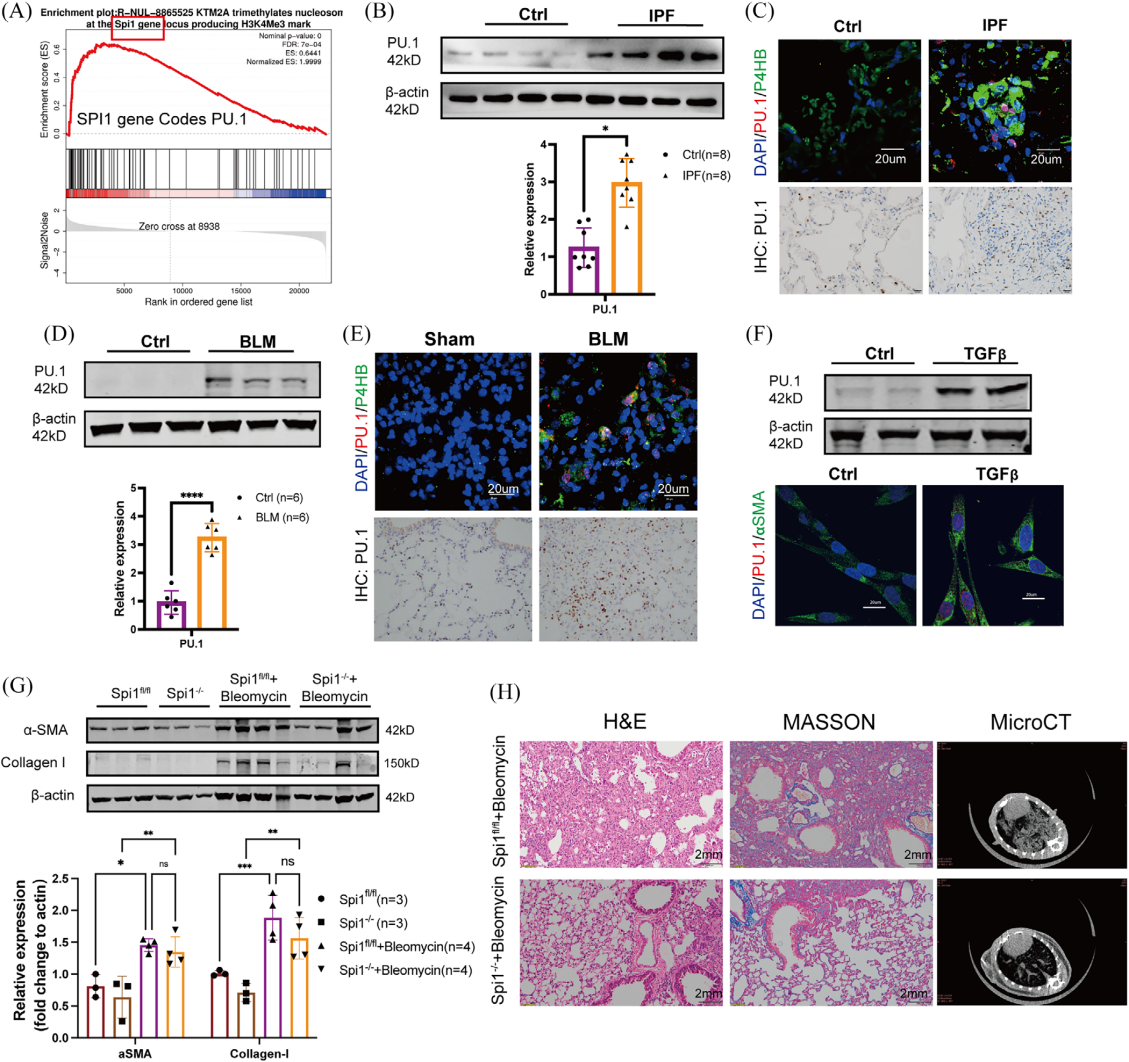
Transcription factor PU.1 was a target of KMT2A. (A) Reactome enrichment of primary fibroblasts from mice with fibrotic lung. (B) Western blotting images and relative expression of PU.1 in human primary lung fibroblast. (C) Immunofluorescent staining of P4HB and KMT2A and immunohistochemical staining of KMT2A in IPF tissue sections. (D) Western blotting images and relative expression of PU.1 in mice primary lung fibroblast. (E) Immunofluorescent staining of P4HB and KMT2A and immunohistochemical staining of KMT2A in mice tissue sections. (F) Western blotting images and relative expression of PU.1 in TGFβ‐stimulated MRC‐5 cells. Immunofluorescent staining of PU.1 in TGFβ‐stimulated MRC‐5 cells. (G) Western blotting images and relative expression of α‐SMA and Collagen I were reduced in lung tissues of Spi1^−/−^ mice treated with bleomycin. (H) H&E, MASSON and MicroCT showed fibrotic area in Spi1^−/−^ mice treated with bleomycin. *p < .05, **p < .01, ***p < .001, ****p < .0001.

In primary fibroblasts from mice with fibrotic lung, we discovered through transcriptome sequencing that PU.1 could be a downstream target of KMT2A (Figure 3A). PU.1 is a member of the ETS family of transcription factors, encoded by the SPI1 gene.^19^ Recent studies have found that elevated expression of PU.1 is associated with the development of various fibrotic diseases.^15^, ^16^ We first validated the expression of PU.1 in IPF‐derived primary fibroblasts and a classical TGFβ cell model. PU.1 levels were significantly up‐regulated in primary cells, as well as in the classical TGF cell model (Figure 3B,F). Immunohistochemical staining of IPF tissue sections revealed an increased number of PU.1‐positive cells in the IPF tissues. Subsequent immunofluorescence co‐localisation of IPF tissue sections showed elevated PU.1 expression levels in the nuclei of fibroblasts compared with the control group (Figure 3C). Additionally, cell immunofluorescence showed increased PU.1 expression in the TGF cell model, localised in the nuclei of fibroblasts (Figure 3F). In BLM‐induced pulmonary fibrosis mice, we observed a significant up‐regulation of PU.1 levels through tissue paraffin sections and immunofluorescence co‐localisation. PU.1 was notably elevated and localised in the nuclei of P4HB‐positive cells (Figure 3E). PU.1 levels were significantly up‐regulated in both BLM‐treated mouse lung primary cells (Figure 3D). In vitro, PU.1 was predicted to bind with promoters of pro‐fibrotic genes including ACTA2 (Actin Alpha 2, Smooth Muscle Actin), COL1A1 (Collagen I Alpha 1) and COL1A2 (Collagen I Alpha 2) by use of JASPER (Figure S1). In vivo, we conducted conditional knockout mice of SPI1 gene (coding PU.1) and performed bleomycin‐induced lung fibrosis model (Figure S2). Western blotting results indicated that the expression of Collagen I and α‐SMA was down‐regulated in the lungs of Spi1^−/−^ mice but did not reach statistical significance (Figure 3G). However, H&E and Masson trichrome staining revealed that fibrotic area was reduced in Spi1^−/−^ mice treated with bleomycin (Figure 3H).

### 3.4 Exogenous intervention in KMT2A expression impacts PU.1 levels

**FIGURE 4:**
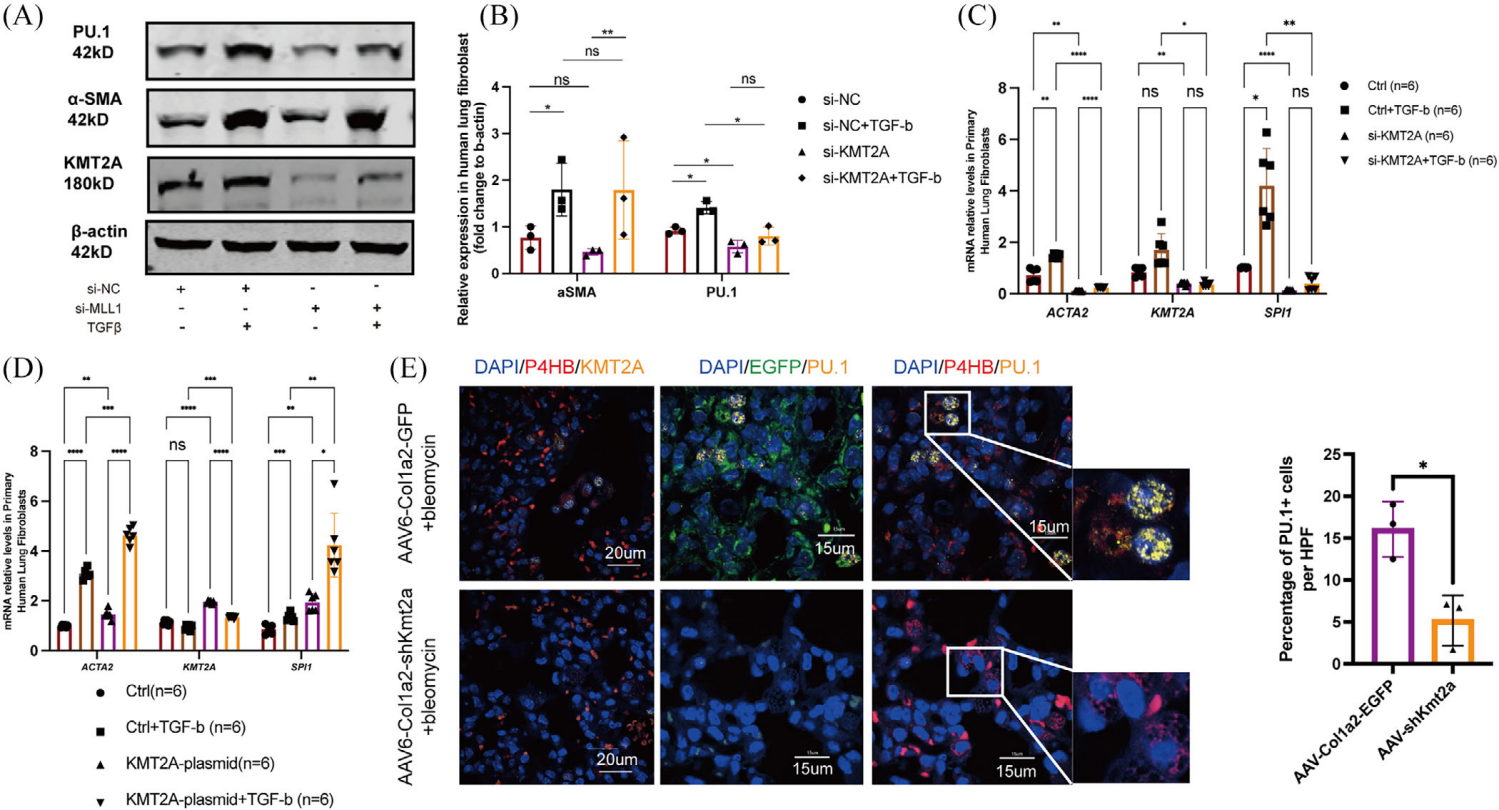
Exogenous intervention in KMT2A expression impacts PU.1 levels. (A) Western blotting images and relative expression of α‐SMA, PU.1 and KMT2A in siRNA‐KMT2A‐transfected MRC‐5 cells. (B) Relative expression and statistical results of α‐SMA, PU.1 and KMT2A in siRNA‐KMT2A‐transfected MRC‐5 cells. (C) Quantitative realtime PCR of α‐SMA, PU.1 and KMT2A mRNA levels in siRNA‐KMT2A‐transfected MRC‐5 cells. (D) Quantitative realtime PCR of α‐SMA, PU.1 and KMT2A mRNA levels in KMT2A‐plasmid‐transfected MRC‐5 cells. (E) Immunofluorescent staining of PU.1 and percentage of PU.1‐positive fibroblast in AAV6‐Col1a2‐shKmt2a‐infected mice lung tissues. *p < .05, **p < .01, ***p < .001, ****p < .0001. siNC, non‐targeting control siRNA; siKMT2A, siRNA targeting KMT2A gene.

To validate the regulation of PU.1 expression in fibroblasts by KMT2A, we first intervened in the expression of KMT2A using siRNA in MRC‐5 cells. At the protein level, PU.1 expression significantly decreased following the down‐regulation of KMT2A, while the expression of α‐SMA in KMT2A knockdown cells showed a downward trend but without statistical significance (Figure 4A,B). qPCR results indicated that after si‐KMT2A knockdown, the transcription levels of ACTA2 and SPI1 were significantly reduced (Figure 4C). Overexpression of KMT2A in fibroblasts led to an increase in PU.1 mRNA transcription, and further TGFβ stimulation led to an additional activation of ACTA2 and SPI1 gene transcription (Figure 4D). In vivo, immunofluorescent staining showed a decreased number of PU.1‐positive cells in the lung tissues of BLM mice infected with AAV6‐Col1a2‐shKmt2a (Figure 4E), indicating the regulatory role of KMT2A on PU.1.

### 3.5 KMT2A regulates PU.1 expression by modulating promoter H3K4me3

**FIGURE 5:**
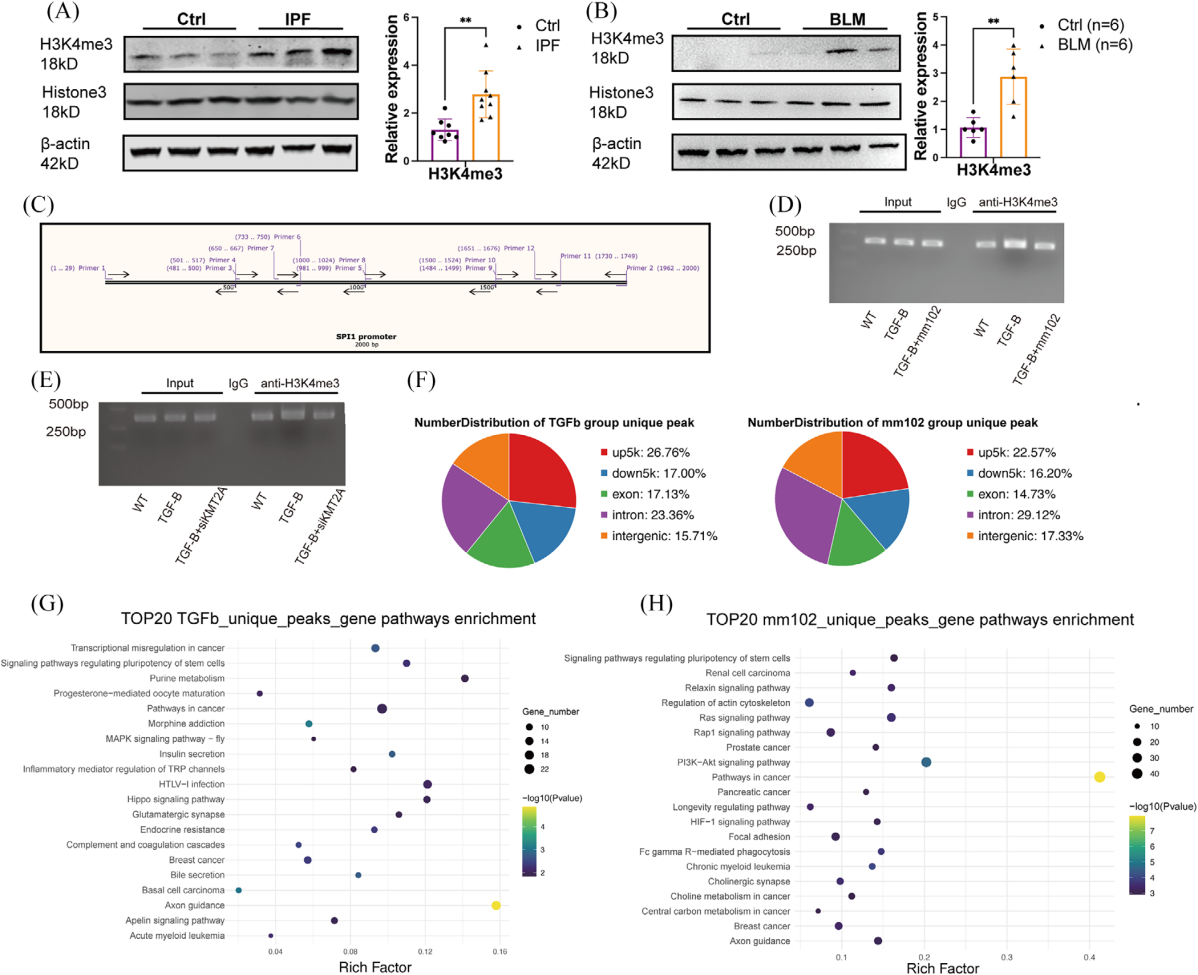
KMT2A regulates PU.1 expression by modulating promoter H3K4me3. (A) Western blotting images and relative expression of H3K4me3 in human lung primary fibroblast. (B) Western blotting images and relative expression of H3K4me3 in mice lung primary fibroblast. (C) Designing of CUT&RUN PCR primers. (D) Agarose gel blotting of PCR products showed H3K4me3 modification is significantly enriched in the region upstream of −350 bp from the transcription start site (TSS) of SPI1, and KMT2A inhibitor mm102 reduced H3K4me3 modification. (E) Agarose gel blotting of PCR products showed H3K4me3 modification enriched in the region upstream of −350 bp from the TSS of SPI1 was reduced by siRNA targeting KMT2A. (F) CUT&Tag peak analysis of TGFβ‐stimulated cells (TGFb group) showed peak mainly at upstream 5 kb region from TSS. Analysis of TGFβ+mm102‐treated cells (mm102 group) showed peak mainly at intron sites. (G) CUT&Tag top 20 peak gene pathway enrichment in TGFβ‐stimulated MRC‐5 cells. (H) CUT&Tag top 20 peak gene pathway enrichment in TGFβ+mm102‐treated MRC‐5 cells. *p < .05, **p < .01, ***p < .001, ****p < .0001.

To investigate the specific mechanism by which KMT2A regulates PU.1 expression, we examined the levels of histone modifications. The results demonstrated that in both IPF patient tissues and model mouse fibroblasts, the levels of H3K4me3 modification were significantly up‐regulated (Figure 5A,B). The CUT&RUN results indicated that the H3K4me3 modification is significantly enriched in the region upstream of −350 bp from the transcription start site (TSS) of SPI1 (which encodes PU.1) (primers 12 and 2). The small molecule inhibitor mm102 is a mimetic of KMT2A that inhibits the function of KMT2A transcription complex. Under conditions of equal cell numbers and DNA product concentrations, treatment with mm102 resulted in a reduction of H3K4me3 modification in this region (Figure 5C,D). Also, knocking‐down KMT2A by siRNA led to reduction of H3K4me3 modification in this region (Figure 5E). The CUT&Tag sequencing results revealed that 26.76% of the H3K4me3 modification is enriched in the upstream regulatory regions of genes in TGFβ‐stimulated cells (Figure 5F). In TGFβ‐stimulated cells, H3K4me3 was enriched in signalling pathways such as transcriptional misregulation in cancer, signalling pathways regulating pluripotency of stem cells and Hippo signalling pathway. When the transcriptional complex function of KMT2A is inhibited by mm102 in fibrotic fibroblasts, the changes in H3K4me3 modification are enriched in pathways related to aberrant transcription of oncogenes, genes associated with renal cell carcinoma, breast cancer and basal cell carcinoma (Figure 5G,H). These findings suggest that epigenetic modifications in fibrotic fibroblasts are involved in various aspects such as cellular oncogenesis and intercellular communication.

### 3.6 The KMT2A inhibitor mm102 attenuated bleomycin‐induced pulmonary fibrosis

**FIGURE 6:**
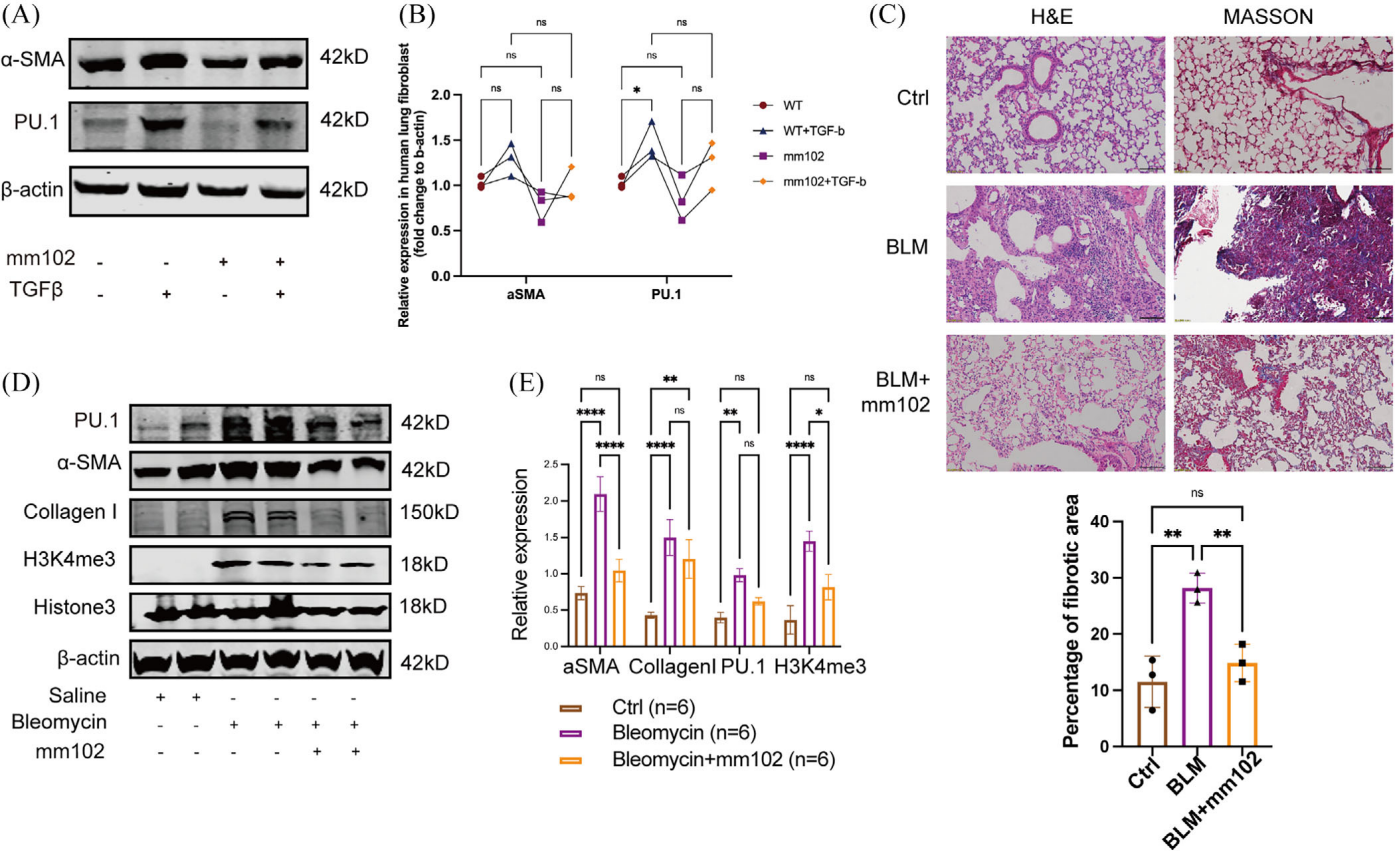
KMT2A forms a complex with WDR5 to jointly regulate PU.1 expression. (A) Western blotting images and relative expression of α‐SMA and PU.1 in mm102‐treated MRC‐5 cells. (B) Relative expression and statistical results of α‐SMA and PU.1 in siRNA‐KMT2A‐transfected MRC‐5 cells. (C) H&E and MASSON staining in mice lung tissue treated with bleomycin and mm102. Percentage of fibrotic area in mice of each group was shown. Mice treated with mm102 revealed less fibrotic area in the tissue. (D) Western blotting images of α‐SMA, PU.1, Collagen I and H3K4me3 in mm102‐treated bleomycin‐induced mice model. (E) Relative expression of α‐SMA, PU.1, Collagen I and H3K4me3 in mm102‐treated bleomycin‐induced mice model showed decreased extracellular matrix production. *p < .05, **p < .01, ***p < .001, ****p < .0001.

KMT2A, when functioning as a methyltransferase, can be cleaved into two active subunits: the N‐terminal and C‐terminal. The N‐terminal subunit localises to histones by interacting with transcriptional coactivators, while the C‐terminal subunit forms an enzymatically active catalytic complex with the highly conserved proteins WDR5, RBBP5, DPY30 and ASH2L. Together, these components collaborate to activate gene transcription.^20^, ^21^, ^22^ The V‐shaped cleft structure of the WDR5 protein and the valine–aspartate–valine motif of RBBP5 are essential for the function of the C‐terminal subunit of KMT2A.^23^ The small molecule inhibitor mm102 can competitively bind to WDR5, thereby inhibiting the function of the transcription complex. Cells treated with mm102 showed a downward trend in PU.1 expression and a corresponding change in the expression of α‐SMA (Figure 6A,B). To validate the specific role of inhibiting the interaction between KMT2A and WDR5 in the development of pulmonary fibrosis, we administered the KMT2A competitive inhibitor mm102 as an intervention treatment in a pulmonary fibrosis mouse model. Histological analysis of lung tissue slices with H&E and Masson staining revealed that the saline group had normal lung tissue architecture with no signs of inflammatory cell infiltration or collagen fibre proliferation. In contrast, the BLM group showed disrupted lung tissue architecture, collapsed and fused alveoli and evident fibrosis with collagen fibre proliferation. However, the mm102‐treated group exhibited reduced inflammatory infiltration and decreased collagen synthesis compared with the BLM group (Figure 6C). Western blot analysis showed a significant up‐regulation of Collagen I and α‐SMA expression in the BLM group, while mm102 treatment resulted in decreased α‐SMA and H3K4me3 levels, along with a downward trend in PU.1 and Collagen I expression (Figure 6D,E). These results suggested that inhibiting KMT2A function could be a potential therapeutic approach for treating pulmonary fibrosis.

## 4 DISCUSSION

We analysed IPF microarray data from the GEO database and found that the histone methyltransferase KMT2A is up‐regulated in IPF. We observed for the first time that high expression of KMT2A is localised in lung fibroblasts. Additionally, by using adeno‐associated viruses with fibroblast‐specific promoters to knockdown KMT2A in mouse lung tissues, we found that bleomycin‐induced fibrosis was alleviated, suggesting that KMT2A in fibroblasts may play a role in lung fibrosis.

In the pathogenesis of pulmonary fibrosis, histone modifications are recognised as crucial epigenetic mechanisms, with some studies already reporting their involvement. Recent research has shown that the histone acetyltransferase Mof promotes the transcription of genes such as ACTA2, COL1A2 and SURVIVIN in fibroblasts through H4K16ac modifications, thereby increasing extracellular matrix synthesis, facilitating cellular phenotype transformation and advancing pulmonary fibrosis.^24^ Additionally, members of the HDAC family, including HDAC1, HDAC3 and HDAC8, which are highly expressed in lung tissues, have been found to suppress epithelial–mesenchymal transition and fibroblast invasiveness, promote extracellular matrix synthesis and participate in inflammatory responses and cell cycle regulation, all contributing to pro‐fibrotic functions. Notably, pan‐HDAC inhibitors have been shown to significantly treat bleomycin‐induced pulmonary fibrosis.^25^, ^26^, ^27^ In this study, we report for the first time that the expression of the histone methyltransferase KMT2A is significantly up‐regulated in fibroblasts from both IPF patients and bleomycin‐induced pulmonary fibrosis mouse models. While the role of KMT2A in fibrosis progression has been documented in liver and kidney fibrosis, its involvement in pulmonary fibrosis has not been previously explored.

Recently, the role of KMT2A in fibrotic diseases and its potential therapeutic targets have garnered increasing attention, with its downstream targets exhibiting diverse functions. In a unilateral ureteral obstruction‐induced obstructive nephritis model, the KMT2A and H3K79me3‐binding protein menin complex up‐regulates the expression of α‐SMA and fibronectin in fibroblasts, thereby promoting renal fibrosis.^28^ In diabetic nephropathy, KMT2A regulates the H3K4me3 modification of the ZEB1 gene promoter in renal tubular epithelial cells, activating its expression.^29^ In an alcoholic liver disease model, studies have shown KMT2A targets pro‐fibrotic genes, including the proto‐oncogene c‐JUN and fibroblast growth factor binding protein 3.^30^ Additionally, KMT2A can target the expression of Caveolin‐1 in liver endothelial cells, playing a role in LPS‐induced liver fibrosis.^31^ This study figured out transcription factor PU.1 might be the target.

PU.1 comprises four functional domains: the ETS domain, which binds to gene promoters with the 5′‐GGAA‐3′ DNA sequence rich in ‐AT‐; the N‐terminal acidic domain and the glutamine‐rich domain, both of which possess transcriptional activity; and the PEST domain, which is involved in protein–protein interactions.^32^, ^33^ In mice, specific knockout of PU.1 in hepatic stellate cells inhibits their activation, reducing liver fibrosis. Conversely, exogenous expression of PU.1 can promote oxidative stress and inflammation by inhibiting Sirt1, leading to extracellular matrix production and hepatic lobule remodelling, which results in fibrosis.^34^ Also, selective PU.1 inhibitors have been shown to alleviate liver inflammation and insulin resistance, significantly reducing the extent of liver fibrosis.^35^ These studies suggest that PU.1 plays a role in mediating organ fibrosis, with its functions potentially involving multiple signalling pathways related to cell metabolism, proliferation and inflammation. In this study, specific knockout of PU.1 in fibroblasts can reduce mice's susceptibility to bleomycin and alleviate lung fibrosis. We found that KMT2A can enhance the protein expression level of PU.1 and increasing the H3K4me3 modification in the PU.1 gene promoter region, specifically the upstream −0.35 kb region of the TSS. However, the molecular function of PU.1 in fibrogenesis needs further exploration.

Previous studies have shown that KMT2A regulates genes relying heavily on the participation of its complex‐associated proteins. In research on renal ischemia–reperfusion injury in mice, KMT2A, in conjunction with WDR5, up‐regulates the expression of p16INK4a. Knockdown of KMT2A and WDR5 both result in suppressed p16INK4a expression. Moreover, using a competitive inhibitor of KMT2A to prevent complex formation in animal models significantly improved lung fibrosis and injury in mice.^36^ Another component of the KMT2A complex, RBBP5, can undergo phosphorylation at the S350 serine residue. Phosphorylated RBBP5 not only stabilises DPY30 expression but also enhances the methyltransferase activity of KMT2A.^36^ Besides its role in facilitating H3K4 methylation through the KMT2A complex, DPY30 itself regulates the differentiation and proliferation of haematopoietic stem cells and progenitor cells, and it promotes cell growth in leukaemia cells induced by KMT2A fusion genes.^37^ These findings suggest that when investigating regulatory mechanisms of KMT2A on PU.1, the involvement of other components of the KMT2A complex should also be considered. Furthermore, the role of the KMT2A complex in regulating other fibroblast functions warrants further exploration. In this study, competitive inhibitors of KMT2A could reduce PU.1 expression by inhibiting KMT2A complex, and showed therapeutic effects in bleomycin‐induced pulmonary fibrosis in mice. Further exploration of downstream targets regulating the fibroblast phenotype conversion by KMT2A revealed that changes in H3K4me3 modification was enriched in pathways related to oncogene aberrant transcription, breast cancer‐associated genes and basal cell carcinoma‐associated genes, providing insights for future research.

This study has several limitations.The AAV‐treated model is not a therapeutic model but a prophylactic one, and further research is needed to validate the target potential of KMT2A. Additionally, we were unable to use a large number of primary fibroblasts for all cell experiments due to technical limitations, and we instead used an embryonically derived cell line. Moreover, other targets regulated by KMT2A were not investigated in this study, and we plan to explore them further in future research.

In summary, we have validated that KMT2A modifies the H3K4me3 on the PU.1 gene promoter region, and treatment with KMT2A inhibitor can alleviate bleomycin‐induced pulmonary fibrosis in mice, indicating the potential of KMT2A to be therapeutic target. Future research will focus on further exploring interactions between KMT2A and other proteins and identifying additional targets regulated by KMT2A in fibroblast phenotype conversion.

## AUTHOR CONTRIBUTIONS

Wenting Lyu: Data curation, Formal analysis, Unvestigation, Methodology, Validation, Visualization, Writing original draft, Writing and review. Hui Wang, Tong Ji: Formal analysis, Validation. Ling Liu, Guanning Zhong, Naihui Wan, Suwan Chen: Formal analysis, Validation. Jingyu Chen, Hourong Cai, Hongyang Xu: Resources. Dongjin Wang: Conceptualization, Funding acquisition, Supervision. Jinghong Dai: Conceptualization, Data curation, Funding acquisition, Project adiministration, Supervision, Writing and review.

## ETHICS STATEMENT

The authors have nothing to report.

## Supporting information



Supporting Information

## Data Availability

Experimental data are available as supplementary materials. The CUT&Tag sequencing data would be available upon reasonable request to the corresponding author.
