# Supplementary material for: Histone methyltransferase KMT2A promotes pulmonary fibrogenesis via targeting pro‐fibrotic factor PU.1 in fibroblasts
Source: Clin Transl Med. 2025 Jan 30;15(2):e70217. doi: 10.1002/ctm2.70217 (PMC11782969; doi:10.1002/ctm2.70217)
Supplement: Supplementary file 1 — Supporting Information [file CTM2-15-e70217-s001.docx]

**
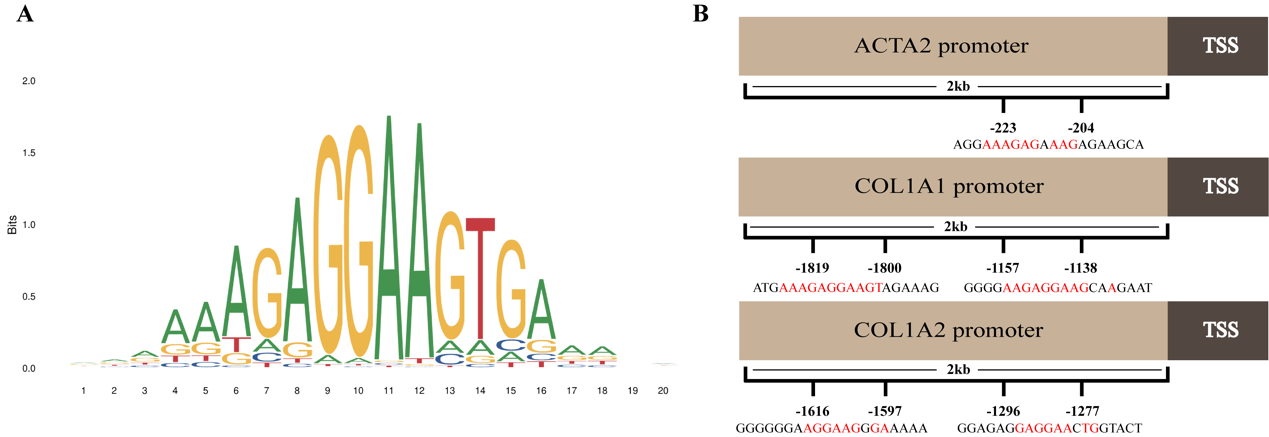
**

**Figure S1 Prediction of PU.1 binding sites using JASPAR**

(A) The canonical binding motif of PU.1. (B) The binding sites of PU.1 in the promoter sequences of ACTA2, COL1A1, and COL1A2.


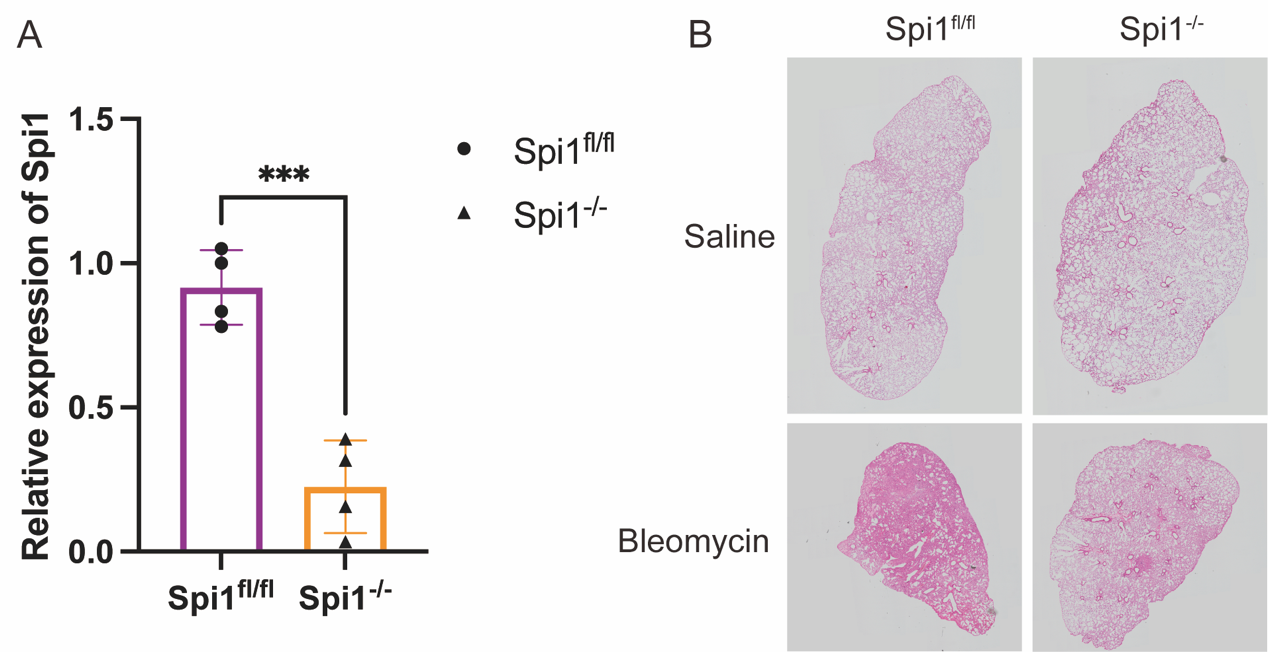


**Figure S2 Validation of Spi1 knock-out**

(A) Quantitative realtime PCR of Spi1 in cKO mice-derived primary fibroblasts showed specific knock-out of SPI1 in fibroblast. (B) The overview of H&E staining on cKO mice lung tissues. *p<0.05, **p<0.01, ***p<0.001, ****p<0.0001.
